# Bias and Evolution of the Mutationally Accessible Phenotypic Space in a Developmental System

**DOI:** 10.1371/journal.pgen.1000877

**Published:** 2010-03-12

**Authors:** Christian Braendle, Charles F. Baer, Marie-Anne Félix

**Affiliations:** 1 Institut Jacques Monod, CNRS–Université Paris Diderot, Paris, France; 2 Department of Biology, University of Florida, Gainesville, Florida, United States of America; Stanford University School of Medicine, United States of America

## Abstract

Genetic and developmental architecture may bias the mutationally available phenotypic spectrum. Although such asymmetries in the introduction of variation may influence possible evolutionary trajectories, we lack quantitative characterization of biases in mutationally inducible phenotypic variation, their genotype-dependence, and their underlying molecular and developmental causes. Here we quantify the mutationally accessible phenotypic spectrum of the vulval developmental system using mutation accumulation (MA) lines derived from four wild isolates of the nematodes *Caenorhabditis elegans* and *C. briggsae*. The results confirm that on average, spontaneous mutations degrade developmental precision, with MA lines showing a low, yet consistently increased, proportion of developmental defects and variants. This result indicates strong purifying selection acting to maintain an invariant vulval phenotype. Both developmental system and genotype significantly bias the spectrum of mutationally inducible phenotypic variants. First, irrespective of genotype, there is a developmental bias, such that certain phenotypic variants are commonly induced by MA, while others are very rarely or never induced. Second, we found that both the *degree* and *spectrum* of mutationally accessible phenotypic variation are genotype-dependent. Overall, *C. briggsae* MA lines exhibited a two-fold higher decline in precision than the *C. elegans* MA lines. Moreover, the propensity to generate specific developmental variants depended on the genetic background. We show that such genotype-specific developmental biases are likely due to cryptic quantitative variation in activities of underlying molecular cascades. This analysis allowed us to identify the mutationally most sensitive elements of the vulval developmental system, which may indicate axes of potential evolutionary variation. Consistent with this scenario, we found that evolutionary trends in the vulval system concern the phenotypic characters that are most easily affected by mutation. This study provides an empirical assessment of developmental bias and the evolution of mutationally accessible phenotypes and supports the notion that such bias may influence the directions of evolutionary change.

## Author Summary

Random mutation does not generate random phenotypic variation because genetic and developmental architecture may constrain and bias the mutationally inducible phenotypic spectrum. Understanding such biases in the introduction of phenotypic variation is thus essential to reveal which phenotypes can ultimately be explored and selected through evolution. Here we used lines which had accumulated spontaneous random mutation over 250 generations starting from four distinct wild isolates of the nematode species *C. briggsae* and *C. elegans*, to study how a developmental system—vulval cell fate patterning—responds to mutational perturbations. We show that developmental defects and variants increase upon mutation accumulation in lines derived from all four isolates. However, some mutationally induced phenotypic variants occur more frequently than others, and the degree and spectrum of developmental variation further differed between isolates. These results illustrate how the phenotypic spectrum induced by random mutation can be biased due to both developmental system features and variation in the genetic background. Moreover, the mutationally most sensitive phenotypic characters are the ones that show most evolutionary variation among closely related species. These observations show how random mutation translates into a biased, limited range of phenotypes—a phenomenon likely impacting possible trajectories of phenotypic evolution.

## Introduction

A principal quest in biology is to disentangle the relative contribution and interplay of mutational versus selective forces in the evolutionary process [1]. While biological research is predominated by the search for adaptive explanation underlying phenotypic evolution, it is also of critical importance to study how the mutational process alone produces phenotypic variation. Such studies indicate which phenotypic space can actually be explored by mutation to generate variation for selection to act upon. The mutationally inducible phenotypic spectrum is thus the fundamentally limiting force constraining and biasing potential evolutionary trajectories of the phenotype.

Importantly, the mutational spectrum is *multidimensional* and *quantitative* in character, where certain regions of the phenotypic space may be easier to reach by mutation than others. In quantitative genetic terms, the mutational variance *V_M_* of the phenotype represents the amount of variation introduced into the population by mutation each generation and can be extended to a multidimensional phenotypic space, theoretically the **M** matrix of mutational variance-covariance between phenotypic traits [2]–[4]. The structure of the mutationally accessible space can be best determined through the use of mutation accumulation (MA) lines, where mutations are allowed to accumulate for many generations with minimal selection [5]. Although the importance of the multivariate mutational process is well-appreciated theoretically [6],[7], empirical data are limited and most studies have focused on complex, composite traits, particularly life-history traits [8]–[10]. To our knowledge, no study has attempted to characterize the multivariate mutational structure of a developmental system.

### Developmental bias and evolution

It is evident that the genotype-phenotype map, encompassing organismal development, is highly non-linear, so that random mutation does not result in random phenotypic variation. For example, mutation may induce plentiful phenotypic variation for one trait but none for another. In the extreme case there is an absolute bias, so that certain phenotypes are impossible to generate though mutationally induced developmental changes, i.e. there is a developmental constraint [11],[12]. The phenomenon of developmental bias can be thought of as milder, relative constraint, where random mutational (or environmental) effects translate preferably into certain phenotypes [13]–[15]. Differences in such bias may be primarily quantitative and can be expressed as different probabilities of generating a given phenotypic spectrum upon random perturbation.

There is circumstantial evidence that developmental bias is common [13], [15]–[19]. In addition, experimental evidence suggests that genetic and developmental architecture bias the production of phenotypic variation. For example, repeated instances of parallel evolution indicate that evolution may follow a limited range of pathways [e.g. 20,21]. However, identifying the relative contribution of mutational versus selective forces in these comparative analyses remains challenging. Recent tests using experimental evolution approaches provide direct evidence on how genetic architecture may bias molecular variation made available to selection [22].

Overall, very few studies [e.g. 23] have quantified the inducible spectrum of phenotypic variation to evaluate whether “intrinsic” tendencies may influence the direction of phenotypic evolution. In general, as pointed out by Yampolsky & Stoltzfus (2001) there is little research focusing on experimental characterization of the *spectrum of spontaneous variation* and the underlying causes of molecular and developmental causes of any observed biases, which would allow testing the hypothesis that biases in the introduction of variation have influenced evolutionary patterns of the examined traits.

### Genotype-dependence of developmental mutability

The mutational architecture may itself evolve, i.e. the regions of phenotypic space reached by mutation differ among genotypes. In other words, developmental bias is genotype-dependent. The inducible phenotypic spectrum for a given genotype has been referred to as “phenotypic neighbourhood” [24] or “local bias” [17]. Such evolutionary variation in mutational properties may be characterized by comparative quantitative analyses of mutation accumulation (MA) lines started from multiple distinct genotypes. Such studies show that mutational parameters may vary substantially between taxa and/or between genotypes of a single species [25]–[27]. We previously showed that mutational damage accumulates about twice as fast in *C. briggsae* as in *C. elegans* for lifetime reproductive output (≈“fitness”) [25],[28], body size [29], and at dinucleotide microsatellites [30]. These results reveal evolution of quantitative biases in the production of phenotypic variation (which could be due to evolution of mutation rates), but the underlying developmental and molecular causes of biases in the examined traits are so far unknown. To quantify and evaluate the significance of developmental bias and its genotype-dependence, analogous studies need to be carried out in simple, tractable developmental systems.

### The study system: *Caenorhabditis* vulval cell fate patterning

*C. elegans* vulval cell fate patterning is a model system for the study of intercellular signalling events [31] and has also served to study developmental robustness, cryptic variation and evolution [32]–[35].

**Figure 1:**
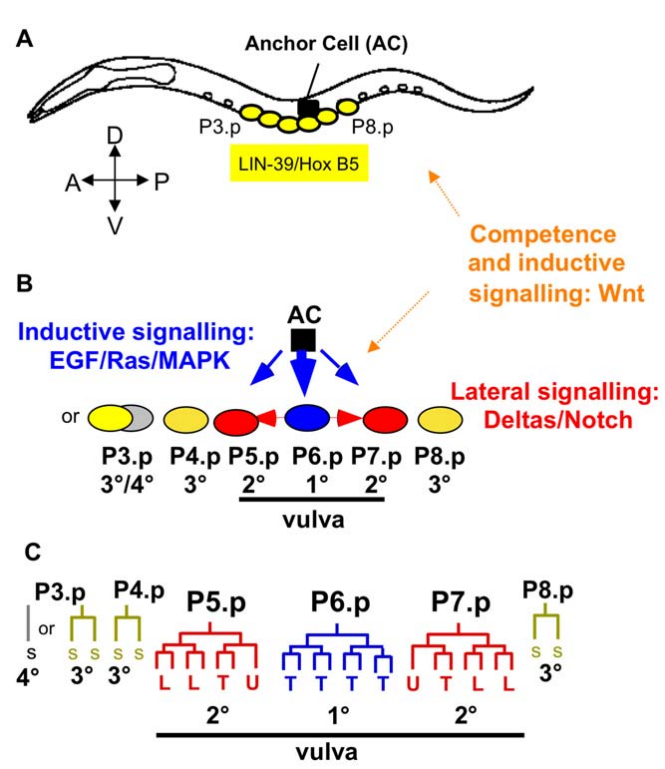
*Caenorhabditis elegans* vulval cell fate patterning. The different cell fates of P3.p to P8.p are characterized by their cell lineage, i.e. number and orientation of cell divisions. Only three precursor cells, P5.p, P6.p and P7.p, adopt a vulval fate: P6.p adopts the central 1° vulval fate, and P5.p and P7.p the outer 2° vulval fate. P4.p and P8.p adopt a non-vulval 3° fate. P3.p shows high variability in its cell fate: it may divide once (3° fate) or it directly fuses with the syncytium without division (4° fate). For the reference isolate N2, the ratio of individuals adopting the 3° versus the 4° fate is approximately 1:1 [37],[68]. The canonical *C. elegans* pattern for P3.p to P8.p is thus defined as follows: 3°/4°−3°−2°−1°−2°−3°. The vulval cell fate pattern is conserved in *Caenorhabditis*. The lineages and competence properties of P3.p, P4.p and P8.p, however, may vary within and between species. Specifically, P3.p divides less frequently and is less competent (to adopt a vulval fate) in *C. briggsae*; in addition, P4.p and P8.p do not always divide in *C. briggsae*, i.e. they do sometimes adopt a 4° instead of a 3° fate [34],[37],[38],[57]. (A) L1-L2 stages: Competence establishment and maintenance of the vulval competence group. (B) Early L3 stage: Specification of vulval precursor cell fates involving primarily EGF/Ras/MAPK and Delta/Notch pathways. (C) Late L3 stage: Cell lineages. Each vulval fate corresponds to an invariant cell division pattern executed during the late L3 stage, resulting in a total of 22 vulval cells. The cell lineages of P5.p to P7.p are identical in all *Caenorhabditis* species [38]. Vulval morphogenesis takes place during the L4 stage and the complete vulval organ develops by the final moult to the adult. AC: anchor cell, T: transverse (left-right) division, L: longitudinal (antero-posterior) division, U: undivided, SS: fusion to the epidermal syncytium (hyp7) after single division (3° fate); S: fusion to the syncytium in the L2 stage with no division (4° fate) (3° and 4° fates: non-vulval fates).

The *C. elegans* hermaphrodite vulva develops from a subset of ventral epidermal blast cells, the Pn.p cells. In wild-type animals, three neighbouring cells, P5.p, P6.p and P7.p adopt vulval cell fates in the sequence 2°−1°−2°. Furthermore, three additional Pn.p cells, P3.p, P4.p and P8.p, have the capacity to adopt a vulval cell fate, when one or more cells of P5.p to P7.p are missing [36]. The six cells, P3.p to P8.p, therefore constitute the vulval competence group. During the second and third larval stages, the vulval precursor cells adopt alternative cell fates governed by an intercellular signalling network of Ras, Notch and Wnt pathways (Figure 1). A correct fate pattern of three vulval precursor cells (2°−1°−2°) is required to form a functional vulva. Deviation from this pattern can cause a reduction in offspring number due to impaired egg laying capacity and may further prevent male mating [34].

Vulval cell fate patterning is conserved among *Caenorhabditis* species [37]–[39]: P5.p to P7.p adopt vulval fates with the pattern 2°−1°−2° while all other vulval precursor cells adopt non-vulval fates, either a 3° fate (the Pn.p cell divides once) or a 4° fate (the Pn.p cell fuses early to the epidermal syncytium hyp7 without division). Species, however, may differ in the frequency of 3° versus 4° fate adopted by P3.p, P4.p and P8.p [37] and in the replacement competence of these cells upon laser ablation [38].

We previously quantified the precision of vulval development of (isogenic) *C. elegans* and *C. briggsae* isolates in multiple experimental environments [34],[37]. The results suggest that vulval development is robust to environmental and stochastic perturbations: apparent vulval defects occur in approximately 1 out of 1000 animals [34]. In contrast, developmental defects and variants increased significantly in mutation accumulation lines derived from a single *C. elegans* isolate, N2 [40], thus degrading the precision of vulval cell fate patterning [34]. This result indicates that mutation accumulation represents a feasible approach to quantify largely unbiased, mutationally induced phenotypic variation of this developmental system.

In this study, we examined the variation in mutational responses of the vulval developmental system within and between related species. We used mutation accumulation (MA) lines derived from two *C. briggsae* (HK104 and PB800) and two *C. elegans* (N2 and PB306) wild isolates that had accumulated mutations over approximately 250 generations [25]. We focused on quantifying and characterizing the spectrum of vulval developmental variants induced by spontaneous random mutation to address the following questions:

1) Does *developmental precision decay* upon mutation for all four isolates, and if so, can the *action of natural selection* be inferred by comparison of the degree of precision among wild isolates?

2) Does the vulval developmental system show a bias in its mutational response, i.e. are certain developmental variants more likely to occur than others? Which phenotypic characters of the developmental systems show maximal mutability?

3) Do the *degree* and *spectrum* of mutationally induced developmental variation vary between genotypes, i.e. to what extent is developmental bias genotype-dependent? How does the degree of mutability of a given developmental phenotype relate to its actual evolutionary variation within and between species?

## Results

**Figure 2:**
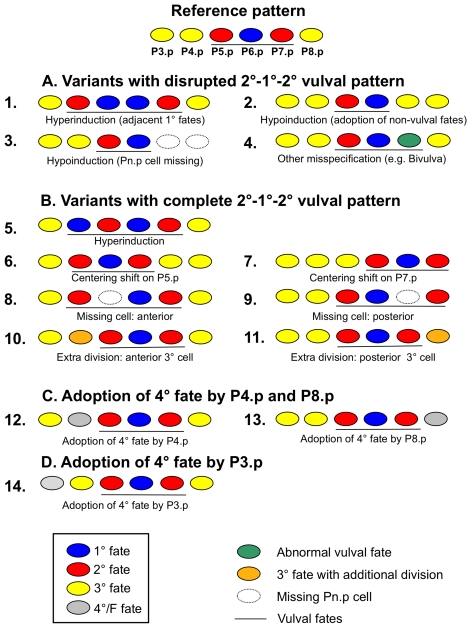
Variant cell fate patterns of vulval precursor cells (P3.p to P8.p). We distinguish three classes of variant vulval patterns in decreasing order of vulva pattern disruption: Variants with disrupted 2°−1°−2° pattern (Class A: “defects”); variants with complete 2°−1°−2° pattern and altered vulval vs. non-vulval fates for the remaining cells (Class B), variants with complete 2°−1°−2° pattern and variable adoption of 3° versus 4° fate by P4.p and P8.p (Class C). 13 non-canonical subcategories of variants are further defined by their deviant cell fate pattern in P(4–8).p (see Material and Methods). Finally we present results on a highly variant trait, P3.p fate: 4° versus 3° (Class D), yet do not include this trait in the analysis of vulva precision. The reference (canonical) pattern for this figure (top) is arbitrarily shown with P3.p adopting a 3° fate. Note that not all variant patterns are mutually exclusive, so that a given individual may adopt multiple variants. (A) Variants with disrupted 2°−1°−2° vulval pattern (Class A). This class groups variant patterns that cause defects in the final vulval structure, likely leading to a reduction in offspring production [34]. (1) Hyperinduction: more than three vulval precursor cells adopt a vulval cell fate (1° or 2° fate), preventing the formation of a complete vulva. For example, P8.p is induced and displaces P7.p progeny from the main invagination. Such cases of hyperinduction are often observed in the presence of an additional anchor cell. (2) Hypoinduction due to adoption of 3° or 4° non-vulval fates: fewer than three cells adopt a vulval cell fate (1° or 2° fate) because of a fate change from vulval to non-vulval. Example: P7.p adopts a 3° non-vulval fate instead of a 2° vulval fate. (3) Hypoinduction due to missing cells: Fewer than three cells adopt a vulval cell fate because one or several Pn.p cells are missing. Example: P7.p and P8.p are missing and only two cells, P5.p and P6.p, adopt vulval cell fates. (4) Misspecification of vulval fates (other than hyper- and hypoinduction): three cells adopt vulval fates but their cell lineages deviate from the canonical pattern. Example: P7.p is misspecified (in green) and adopts the lineage LLTU instead of UTLL. Such a defect in lineage orientation causes a ventral protrusion and is referred to as Bivulva phenotype [69]. Although this specific case of fate misspecification need not always disrupt functionality of the vulva, it eliminates the capacity to mate with males [70]. (B) Variants with complete 2°−1°−2° pattern and altered vulval versus non-vulval fates for the remaining cells (Class B). Such variant patterns do not obviously disrupt the formation of a functional vulval organ; however, whether certain variants negatively impact egg laying or other functions is unclear [34]. (5) Hyperinduction: more than three cells adopt vulval cell fates. Example: P4.p adopts a 2° vulval cell fate instead of a 3° non-vulval cell fate. (6–7) Vulval centering shifts: the three cells adopting vulval fates are shifted to the anterior (centering on P5.p) or posterior (centering on P7.p). Example: P5.p adopts a 1° vulval fate while P4.p and P6.p adopt a 2° fate, with the anchor cell being attached to P5.p progeny. (8–9) Missing cells: One or more vulval precursor cells are missing. Example: P7.p is missing and P8.p adopts a 2° vulval fate instead. Note that in our experiments we could not distinguish whether this variant was due to a missing P7.p or P8.p cell. Therefore, we distinguish only whether one or more anterior cell (P3.p to P5.p) or a posterior cell (P7.p and P8.p) was missing. (10–11) Supernumerary cell divisions: Anterior (P3.p or P4.p) or posterior (P8.p) cells divide more than once, generating three or four cell progeny that fuse with the hypodermis. Example: P4.p (shown in pink) divides twice instead (lineage “ssss” instead of “SS”). (C) Adoption of 3° versus 4° fate of P4.p and P8.p (Class C). This class includes: (12) P4.p adopts the 4° fate or F fate, fusing with the hypodermis without prior division. (13) P8.p adopts the 4° fate. (D) Adoption of 3° versus 4° fate by P3.p (Class D). (14) P3.p adopts the 4° fate (frequent in the wild type).

The canonical vulval cell fate pattern in *C. elegans* and *C. briggsae* ancestral controls is 3°−2°−1°−2°−3° (P4.p to P8.p), whereas the most anterior P3.p cell adopts either a 3° or a 4° fate (Figure 1). The MA lines showed a consistently increased proportion of diverse variants (Figure 2), although the canonical P(4–8).p pattern remained the most frequent. Based on the observed variation in MA lines, we distinguished 13 distinct non-canonical cell fate *variants* deviating from the canonical vulval pattern (Material and Methods; Figure 2 legend). For some tests, these 13 variants were placed into three classes of decreasing order of vulva pattern disruption (A, B, and C). All variants were expressed in proportion of animals adopting the corresponding pattern.

### Mutational decay of developmental precision

#### Proportions of variant vulval cell fate patterns (*p*)

(Table 1) - The frequency of non-canonical vulval variants was very low in the ancestral controls, approximately 0.4% in *C. briggsae* and 0.05% in *C. elegans*, averaged over variants #1–13 and isolates. The tenfold difference in the frequency of variants between the two species was almost entirely due to variants in the adoption of 3° versus 4° fate by P4.p and P8.p (class C, Table 1). Overall, for each of the four tested isolates, defective and other vulval variant patterns were more frequent in MA lines than in ancestral controls.

**Table 1:** Proportion *p* of individuals exhibiting variant vulval phenotypes.

Class A: Variants with disrupted 2°−1°−2° pattern (defects)								
Species	*C. briggsae*				*C. elegans*			
Isolate	HK104		PB800		N2		PB306	
Treatment	Control	MA	Control	MA	Control	MA	Control	MA
1. Hyperinduction	0	0	0	1.91 (1.09)	0	0.76 (0.54)	2.48 (1.89)	1.60 (0.71)
2. Hypoinduction (3° or 4° cell fate)	1.25 (1.22)	3.71 (1.57)	0	8.44 (4.14)	0	1.54 (0.74)	0	0.41 (0.38)
3. Hypoinduction (missing cells)	0	1.92 (1.13)	0	0.77 (0.55)	0	0	0	0.37 (0.37)
4. Other fate misspecification	0	6.29 (5.85)	0	0.79 (0.78)	0	2.75 (0.89)	1.20 (1.21)	1.56 (0.99)
**Total proportion (A)**	1.25 (1.22)	11.92 (6.46)	0	11.90 (6.00)	0	5.05 (1.37)	3.68 (2.92)	3.93 (1.40)

Class B: Variants with complete 2°−1°−2° pattern and altered vulval versus non-vulval fates for the remaining cells								
5. Hyperinduction	0	6.01 (3.83)	0	1.89 (0.92)	0	0.38 (0.37)	0	7.08 (3.78)
**Centering shifts (6+7):**	3.14 (1.82)	14.87 (6.07)	0	7.61 (3.31)	1.27 (1.23)	8.29 (3.61)	0.63 (0.85)	5.05 (2.42)
6. Centering on P5.p	0.63 (0.85)	9.76 (5.35)	0	3.04 (1.50)	1.27 (1.23)	8.29 (3.61)	0.63 (0.85)	4.65 (2.34)
7. Centering on P7.p	2.52 (1.75)	5.11 (3.16)	0	4.57 (2.95)	0	0	0	0.41 (0.38)
**Missing Pn.p cells (8+9):**	0	6.22 (2.67)	1.26 (1.21)	9.12 (3.45)	1.22 (1.19)	4.20 (2.07)	1.27 (1.25)	5.65 (1.92)
8. Anterior cell missing	0	1.46 (1.01)	1.26 (1.21)	3.11 (1.31)	1.22 (1.19)	0.77 (0.73)	1.27 (1.25)	2.04 (0.86)
9. Posterior cell missing	0	4.77 (1.95)	0	6.02 (2.73)	0	3.43 (1.82)	0	3.62 (1.53)
**Extra 3° cell divisions (10+11):**	3.75 (3.67)	4.95 (1.98)	0	1.96 (0.98)	0	1.91 (0.85)	0	3.60 (1.86)
10. Anterior 3° cell	2.50 (2.50)	3.59 (1.60)	0	0.79 (0.52)	0	0.77 (0.52)	0	1.64 (1.08)
1I. Posterior 3° cell	1.25 (1.22)	1.36 (0.74)	0	1.17 (0.65)	0	1.15 (0.65)	0	1.97 (1.16)
**Total proportion (B)**	6.89 (4.15)	32.05 (7.23)	1.26 (1.21)	20.58 (6.19)	2.48 (1.66)	14.77 (5.07)	1.89 (1.63)	21.38 (6.47)

Class C: Adoption of 3° versus 4° fate by P4.p and P8.p								
12. P4.p: 4° fate	13.29 (2.52)	23.25 (4.25)	19.31 (4.79)	41.00 (5.78)	2.54 (1.61)	7.75 (2.34)	3.70 (2.04)	4.33 (1.17)
13. P8.p: 4° fate	16.27 (3.76)	25.63 (4.59)	40.00 (7.05)	32.25 (6.16)	0	0	1.24 (1.21)	1.61 (0.89)

Class D: Adoption of 3° versus 4° fate by P3.p								
14. P3.p: 4° fate	96.38 (0.76)	91.17 (1.65)	96.13 (0.86)	92.97 (0.85)	59.47 (2.30)	60.13 (1.67)	73.39 (1.48)	64.66 (2.12)

Variants and classes are lettered and numbered as in Figure 2. Tabled values are the actual value multiplied by 10^3^, except in Class D, where the value is given in % (multiplied by 10^2^). Standard error of the (line) mean is shown in parentheses. Sample Sizes: HK104 (44 MA lines, 17 control lines), PB800 (53 MA lines, 17 control lines), PB306 (51 MA lines, 17 control lines) and N2 (52 MA lines, 17 control lines). For all MA and control line, 50 individuals were scored for their vulval phenotype.

#### Change in mean frequency of variant vulval cell fate patterns (*R_m_*)

(Table S2) - Summing over the variants #1–13, point estimates of *R_m_*, the rate of change in trait mean frequency, were positive in all four isolates. Mean change values in the two isolates of *C. briggsae* (HK104 *R_m_* = 22.3×10^−5^/gen, PB800 *R_m_* = 19.0×10^−5^) were about twice those of the two *C. elegans* isolates (N2 *R_m_* = 9.3×10^−5^, PB306 *R_m_* = 7.9×10^−5^). The individual *R_m_* values for each deviant pattern were overall positive, indicating that most deviant patterns increased in frequency upon mutation accumulation in all four isolates.

#### Change in the among-line variance (*ΔV*)

(Table S3) - The among-line variance in mean values of *p* increased with mutation. The differences among species, isolates, and variants closely mirrored changes in the mean. Summing over all variants #1–13 (Table S3, classes A–C), point estimates of Δ*V* in the two isolates of *C. briggsae* (HK104 = 11.0×10^−6^, PB800 = 13.5×10^−6^) were about 5X greater than those of the two *C. elegans* isolates (N2 = 1.9×10^−6^, PB306 = 2.9×10^−6^).

### Mutational correlations

Correlations of line means between two categories of non-canonical variant patterns (Class A and B) and two categories of fitness-related traits (*W*, *CV_E,W_*) are reported in Table S4. Given the number of variant categories and examined isolates, these tests are not powerful, but several trends emerged from the pattern of correlations. First, the correlation between class A variants (disrupted 2°−1°−2° pattern, likely resulting in defects) and other variants with complete 2°−1°−2° (class B+C) was positive in all isolates. The strength of the correlation between defects and variants was dependent on the starting genotype but was not species-specific: the correlation was strong and significant in *C. briggsae* PB800 and *C. elegans* PB306, but much weaker in the other isolates of each species. Second, the correlation between fitness traits and variants with complete 2°−1°−2° pattern (class B+C), but not variants with disrupted 2°−1°−2° pattern (class A), was stronger in *C. elegans* than in *C. briggsae*. In particular, the correlation between variant classes B+C and the within-line variance in fitness was uniformly strong and positive in *C. elegans* (∼0.5) and much weaker in *C. briggsae* (not significantly different from zero). The correlation in the VEL N2 lines was less than in the CFB N2 lines (∼0.2; not significantly different from zero). Third, all correlations were uniformly weak in the HK104 isolate of *C. briggsae*, a result we have consistently observed in this isolate [29].

### Comparison of mutational variance (V_M_) and standing genetic variance (V_G_)

To compare the mutational variance (V_M_) for variant vulval phenotypes with the standing genetic variance (V_G_), we analyzed data on developmental precision obtained from 10 *C. briggsae* and 25 *C. elegans* isogenic wild isolates (N_individuals_ = 8′460). Wild isolate data are presented in Table S6, showing the proportion of variants for classes A, B, and C. Point estimates of the variance in line means (*V* *_L̂_*) were very low (∼10^−5^) for class A variants (strongly disrupted vulval patterns, defects) and for the pool of class B + C variant categories, and jackknife 95% confidence limits included zero in both categories in all isolates. Further, when isolates for which multiple estimates of *p* were available were considered, the maximum likelihood estimates for the among-isolate (genetic) component of variance were zero for both categories in both species. Thus, vulval development was highly invariant in both *C. elegans* and *C. briggsae* wild isolates, and most of the variant patterns observed were limited to variants of class C (3° to 4° transformation of P4.p/P8.p), in *C. briggsae*.

### Developmental bias: random mutation induces vulval variants at different frequencies

**Figure 3:**
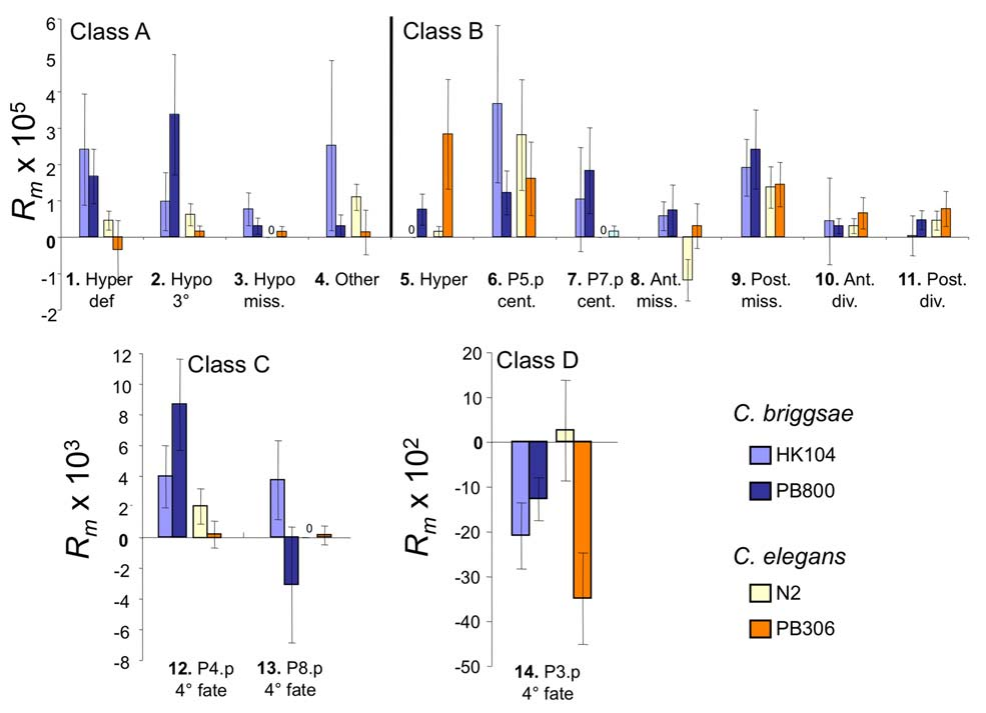
Per-generation change in frequency *R_m_* for variant vulval phenotypes. Mean per-generation change in variant frequency *R_m_* in mutation accumulation lines started from four *C. elegans* and *C. briggsae* isolates (colour-coded). Variants are numbered and placed in four classes (A–D) as in Figure 2. Note the different vertical scales of the graphs. Sample Sizes: HK104 (44 MA lines, 17 control lines), PB800 (53 MA lines, 17 control lines), PB306 (51 MA lines, 17 control lines) and N2 (52 MA lines, 17 control lines). For each MA and control line, 50 individuals were scored for their vulval phenotype. Error bars indicate standard errors of the (line) mean.

Across all four sets of MA lines, the different vulval variant patterns were observed at unequal frequencies (Table 1). Vulval precursor cells adopting a non-vulval 3° fate (P3.p, P4.p and P8.p) showed overall more variability than the cells adopting a vulval cell fate (P5.p to P7.p). Specifically, we found that the developmental phenotype with the highest mutational variance is that already showing high variability in the ancestral controls, i.e. P3.p division frequency (3° versus 4° fate; variant #14; class D) (Table 1 and Figure 3; note change of scale for this variant). The second most common variants concern P4.p and P8.p division frequency (variant #12 and 13; class C). Behind comes a subset of the variant patterns that affect the vulval fates such as centering shifts (class B), hyperinduction (class A or B) or missing precursor cells (class B). Therefore, variants causing likely defects in vulval function (class A) were overall less frequent than variants in classes B or C. That different sub-traits of the vulval developmental system degrade at different rates is further confirmed by the mixed-model analysis of the rate of change in the trait mean frequency *R_m_* (see below).

### Genotype-dependence of developmental mutability

To detect evolutionary variation in the mutability of the vulval developmental system, we tested for an overall interaction between variant vulval phenotype and ancestral genotype in an analysis of variance framework. The mixed-model analysis of the rate of change in the trait mean frequency *R_m_* confirmed a substantial main effect of trait (nominal P<0.0001) and the expected large main effect of species (nominal P<0.002) (Table S5). Thus, the rate of change in mean frequency during mutation accumulation depended on the variant trait and the species. The main effects of isolate (nominal P>0.8) and trait x isolate (nominal P>0.10) were not significant. However, note that several of the most extreme differences in mutational induction of specific vulval variants occurred between the isolates of the same species rather than between species (see below).

Below we report specific examples of genotypic biases in mutationally induced phenotypic variants. Note that because of low frequency of developmental variants and multiple comparisons, the significance level of given comparisons may be poor (the critical experiment-wide 5% significance level for thirteen individual comparisons is P<0.0038).

#### Class A: variants with disrupted 2°−1°−2° vulval pattern

(Table 1 and Figure 3A).

The propensity to generate a specific defective pattern (hypoinduction, variant #2) varied among isolates. This variant was found at the highest frequency in *C. briggsae* PB800-derived MA lines (8/53), and was very rare in *C. elegans* PB306-derived MA lines (1/51 lines: a single individual in the affected line; Table S1) (Fisher's Exact Test, P = 0.036), whereas this variant was never found in the control lines of either of these genotypes. Thus, hypoinduction variants were easier to induce by mutation in *C. briggsae* PB800 than in *C. elegans* PB306.

#### Class B: variants with complete 2°−1°−2° vulval pattern (yet altered vulval vs. non-vulval fates)

(Table 1 and Figure 3B)–Mutational induction of several of these variants showed biases depending on the genotype. Vulval centering shifts on P7.p (variant #7) were most frequent in MA lines of the two *C. briggsae* isolates but never found in MA lines of *C. elegans* N2. Conversely, the induction of excessive vulval cells (hyperinduction, variant #5) occurred more frequently in *C. elegans* N2 than in *C. elegans* PB306 and the two *C. briggsae* lines. Within *C. elegans*, this hyperinduced variant occurred frequently in MA lines derived from PB306 (8/51 MA lines) but not in MA lines derived from N2 (0/52 MA lines) (Fisher's Exact Test, P = 0.005); and this variant was not found in the control lines of either of these two isolates. We further consider this induction variant below.

#### Class C: adoption of 4° versus 3° fate (P4.p, P8.p)

(Table 1 and Figure 3C) - These cell division phenotypes were the most variable traits in *C. briggsae* MA lines. The induction of this variant differed in frequency between the two species: relative to *C. elegans*, *C. briggsae* MA lines as well as ancestral controls showed increased frequency and variability in P4.p and P8.p adopting 4° versus 3° fates. For P8.p, the adoption of the 4° fate never occurred in MA lines derived from *C. elegans* N2, while this variant occurred in MA lines of the other three isolates, with a particularly high frequency for the two *C. briggsae* isolates.

#### Class D: adoption of 4° versus 3° fate (P3.p)

(Table 1 and Figure 3D) - Unlike P(4–8).p, P3.p has a highly variable fate in ancestral controls. In the ancestral controls, P3.p adopted a 4° fate more frequently in *C. briggsae* than in *C. elegans*. After MA, the proportion of P3.p with a 3° fate was increased for *C. briggsae* and *C. elegans* PB306 but not for N2, which showed the highest frequency of P3.p adopting a 3° fate in ancestral controls.

### Causes underlying genotype-dependence of developmental mutability

**Figure 4:**
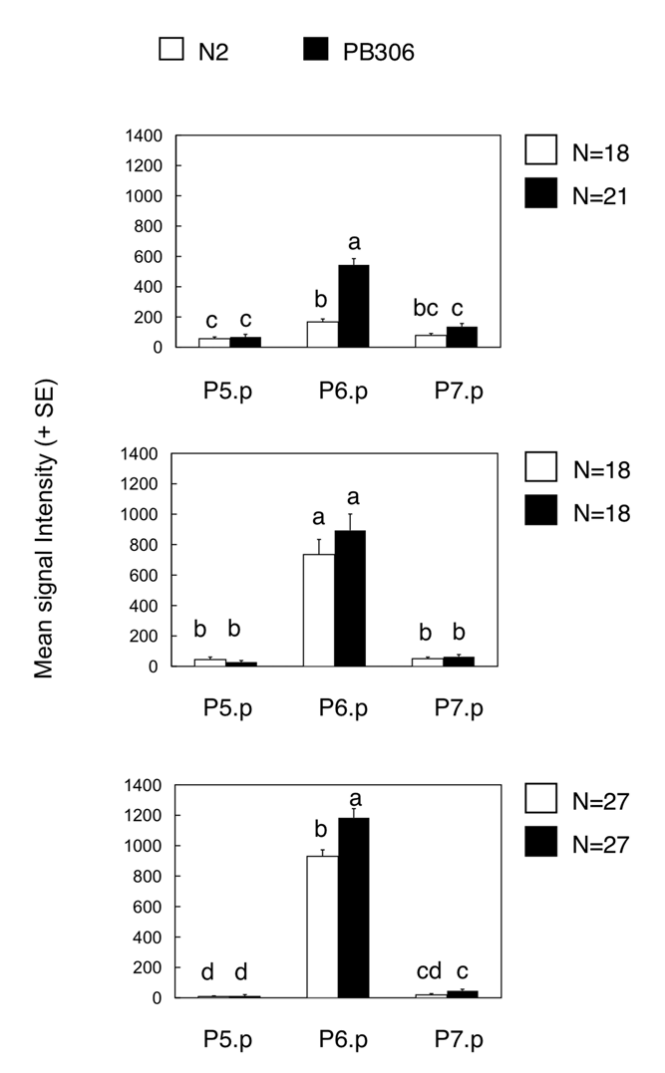
Comparison of Ras pathway activity in isolates of *C. elegans* (N2 versus PB306). Quantification of wild genetic background effects on Ras reporter activity in animals carrying an integrated Ras pathway reporter transgene (*egl-17::cfp-lacZ*), at three distinct developmental stages during vulval induction. Bars labelled with the same letter did not show significant differences in expression levels (Tukey's HSD, P<0.05). Error bars indicate standard error of the mean. For ANOVA results, see Table S7.

The clearest examples of intraspecific variation in the mutational pattern are the hyper- and hypo-induction variants in *C. elegans*: MA lines displayed more hyperinduction variants and less hypoinduction variants in the PB306 isolate compared to the reference isolate N2. One hypothetical scenario to explain the elevated propensity to generate hyperinduced variants upon mutation accumulation in PB306 might be an increased activity of inductive vulval signalling, already present in the ancestral (wild type) genotype. In this scenario, such a difference would rarely be phenotypically expressed in the ancestral genetic background, but become more prevalent in MA lines due to mutational perturbations. To test this hypothesis, we asked whether the activity of the main signalling cascade inducing vulval cell fates, the EGF/RAS/MAPK cascade, was higher in PB306 than in N2. We introgressed an integrated construct containing a transcriptional Ras reporter, *egl-17::cfp* [41], into the two isolates to examine Ras activity levels during the vulval patterning process from mid-L2 to early-L3 stage (see Materials and Methods). Consistent with the hypothesis, PB306 showed a significantly higher Ras pathway activity in the relevant vulval precursor cell, P6.p, during mid-L2 and early L3 stages compared to N2 (Figure 4). Thus, the difference in the mutational accessibility of hyperinduced variants between PB306 and N2 may result through variation in the activity of the Ras pathway, which is phenotypically silent (cryptic) under normal conditions.

## Discussion

### Mutational decay of developmental precision

The developmental system underlying *Caenorhabditis* vulva precursor cell fate patterning was consistently degraded in mutation accumulation (MA) lines derived from all four isolates. In contrast to previously examined traits, such as body size, a quantitative trait varying along a single axis [29],[42], the variation is here practically absent among and within ancestral controls and mutational challenges induce novel variants. Vulval patterning variants almost always had a very low penetrance in a given mutation accumulation line. Many MA lines showed multiple, distinct variants and we never found a line in which a specific variant pattern was fixed.

The observed mutational pattern of small-effect variants may either be explained by non-null mutations in structural genes or mutations in regulatory regions with effects too small to be retained in conventional genetic screens. The core genetic elements of the vulval signalling network amount to approximately 30 genes [31], covering an estimated 150 kb. A conservative estimate of the mutation rate is one mutation per genome per generation in *C. elegans* [43], so that tested MA lines exhibit an average of 250 mutations per genome (100 Mb). Assuming that about a third of the nucleotide sites are susceptible to mutations having some phenotypic effect, the probability of mutating such a site in this category of “identified vulva genes” is 0.125 for a given MA line. This is consistent with the frequency of defects that we observe; however, this estimate is highly speculative, in particular, because we have no information on the distribution of mutational effects at a given locus. Moreover, it appears likely that several of the mutationally induced vulval variants may have been triggered by mutations of genes not directly involved in the vulval signalling network. Diverse developmental mutations primarily affecting body size and shape have the potential to disrupt the spatial and temporal integrity of the vulval induction process [44], and we have observed that many of these mutations (e.g., *dpy, lon, sma, unc*) show diverse low-penetrance vulval variants and defects similar to the ones observed in MA lines.

One consequence of the induction of deviation from an invariant pattern is an increase in the within-line component of variance. We previously demonstrated that the environmental (within-line) component of variance (*V_E_*) consistently increases with mutation accumulation for W, total lifetime fecundity, and body volume in these same lines [45]. We interpreted this result as evidence that spontaneous mutations de-canalize the phenotype, but could not completely rule out the possibility that that result was an artefact of the way in which these data were scaled. In contrast, the increase in vulval variants and defects with MA is most straightforwardly interpreted as an increase in the environmental component of variance, i.e., de-canalization, and it cannot be attributed to scaling. Thus, mutation accumulation increases the sensitivity of the vulval developmental system to stochastic (micro-environmental) perturbations [46].

### Comparison of mutational variance (V_M_) and standing genetic variance (V_G_): strong purifying selection

We calculated an estimate of the standing genetic variance (*V_G_*) for variant vulval phenotypes using data from 25 *C. elegans* and 10 *C. briggsae* wild isolates. At mutation-selection balance in a large population, the ratio of the mutational variance to the standing genetic variance provides an estimate of the strength of purifying selection of mutations affecting the trait, i.e., *S*≈*V_M_/V_G_*, where *S* is the average selection coefficient against a new mutation. Using the point estimate of 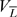 of the wild isolates as a surrogate for *V_G_* and the point estimate of *ΔV* as a surrogate for *V_M_*, the average selection coefficient against mutations affecting Class A variants inferred from the ratio V_M_/V_G_ ( =  S) is on the order of 10% or larger (for *C. briggsae* the point estimate of S = 0.30; for *C. elegans* S = 0.16). Conversely, the ratio V_G_/V_M_ can be interpreted as the “persistence time” of a new mutation, i.e., the expected number of generations the mutation segregates before it is lost [47]. Thus, as expected, new mutations that cause Class A variants segregate for only a very few generations before they are removed by selection (Class A variants in the system are clearly deleterious in laboratory conditions, because they prevent egg-laying and reduce progeny number [34]). By way of comparison to life history traits in the same species, selection coefficients inferred in this way for *W*, body volume, and lifespan are on the order of 1–5% [48],[49]. This result confirms that vulval development is under strong purifying selection to maintain an invariant phenotypic output. The observed selection thus very likely corresponds to the type of stabilizing selection, as defined by Schmalhausen [50], and canalizing selection [51].

Concerning other variant classes, comparison of the genetic variance among wild isolates and after spontaneous mutation accumulation with minimal selection provides indirect evidence of their elimination by selection in natural populations. Especially in class B, the frequency of developmental variants was very low in the four controls as well as in a large set of wild isolates of *C. elegans* and *C. briggsae* covering a much larger range of genetic variation than the MA lines [43],[52] (Table S6). Averaged over variants and species, the ratio V_M_/V_G_ ( = S) of Class B variants is again on the order of 10%, very similar to Class A variants (for *C. briggsae* the point estimate of S = 0.12, for *C. elegans* S≈1). Among the class B variants, variants with vulva centering shifts or missing Pn.p cells (variants #6–9) form a complete vulva due to cell fate regulation among the vulva competence group (cells that can adopt a vulval fate through expression of the *lin-39/Hox* gene [31]). Importantly, this result strongly argues for strong selection against class B variants in natural populations although these variants do not disrupt functionality of the vulval organ and show no fitness effects in the laboratory [34]. By contrast, selection against class C variants appears much weaker (*S* on the order of 0.1%). Class C variants describe variation in non-vulval fates of P4.p and P8.p, which normally do not affect P(5–7).p vulval fates. When adopting the variant pattern (i.e. adoption of the 4° fate), P4.p and P8.p fuse to epidermal syncytium without division in the L2 stage [53], so that the cells lose their competence to respond to late inductive vulval signalling. Nevertheless, these cells may still be able to respond to Wnt or EGF signalling earlier before hypodermal fusion, and thus to replace one of the P(5–7).p cells in the case of co-occurrence of a class B variant.

### Developmental bias: differential mutational accessibility of phenotypic variants

In contrast to classic mutagenesis screens selecting for developmental mutants with high penetrance phenotypes, the screening of the phenotypic spectrum of MA lines is largely unbiased and representative of the phenotypic spectrum induced by spontaneous random mutation. We found that MA induced certain phenotypic variants much more readily than others, demonstrating biases in the mutational accessibility of phenotypic variants. The vulval trait with the highest mutational variance is that already showing high variability in the ancestral controls (P3.p division frequency, variant #14), followed by P4.p and P8.p division frequency (variant #12 and #13; class C). Variants causing likely defects in vulval function (class A) were overall less frequent than variants in classes B or C. In addition, several of these variant patterns have not been found by mutagenesis in the laboratory, presumably because they were too subtle for efficient phenotypic scoring. On the other hand, we did not uncover all possible variant vulval patterns, which suggests that certain of these variants are either fully lethal and could not be propagated in MA lines, or their appearance through mutational effects is too improbable. Such variants include lateral inhibition defects with vulval cells showing adjacent 1° fates as seen in *lin-12/Notch* mutants [54]. Although a fully penetrant loss of lateral inhibition may be lethal, it is interesting that we did not find this variant at low penetrance like other fate pattern variants. This suggests that the mutational target size for this variant (relying on Notch pathway regulation) is small. Taken together, these observations provide clear examples of developmental bias [13]–[15],[18],[19], with certain phenotypic variants being more easily induced by mutation than others.

### Genotype-dependence of developmental mutability

Several results show that biases in the production of vulval variants are genotype-dependent. First, overall rates of mutational decay differ among ancestral controls, most likely due to higher molecular mutation rates in the *C. briggsae* isolates compared to the *C. elegans* isolates [25],[30]. The approximately two-fold greater change in the trait mean in *C. briggsae* was roughly consistent with previous results concerning other traits [28],[29]. Second, we observed differences in the relative mutability of the same canonical pattern to different types of variant pattern. These differences in the mutationally inducible phenotypic spectra may be explained by one of two possible mechanisms. First, the mutation rate at specific loci may vary among wild isolates. For example, a microsatellite repeat present at these loci in some isolates and absent in others may dramatically change mutation rates at the locus [55]. Second, a distinct bias in the developmental system may occur if the internal system variables are slightly offset in some isolates towards the production of a given variant pattern. For example, *C. elegans* PB306 may mutate more frequently to genotypes producing hyperinduction defects if the Ras pathway involved in vulval induction is in average slightly more active in individuals of this isolate (compared to other wild isolates). More mutations of small effect on the system may then tip the balance towards hyperinduction when acting on the *C. elegans* PB306 isolate, and remain silent in other isolates. In this case, the different relative mutability to the hyperinduced phenotype of different starting genotypes may thus depend on cryptic genetic variation causing variation in system parameters, also termed intermediate phenotypes [32].

Apparent cryptic variation in such a quantitative developmental parameter may be confirmed by introgression of mutations or by measurements of signalling pathway activity. A higher Ras pathway activity in the *C. elegans* PB306 isolate is indeed supported by the higher induction index of *let-60(n1046gf/ras, lin-3(n378rf)/egf* mutants and of the *ark-1(sy247lf); gap-1(n1691lf)* double mutant [35]. Our present results using a reporter gene further confirm that the Ras pathway is significantly more active in *C. elegans* PB306 compared to *C. elegans* N2 (Figure 4). This result demonstrates the presence of intraspecific variation in the activity of vulval signalling pathways and agrees with the proposed second mechanism of evolution of the mutational variance through a bias in mutational effects. In the future, the determination of the molecular lesions and their introgression in different genetic backgrounds may definitively answer whether this difference accounts for the increased frequency of hyperinduced variants in PB306.

Mutational and environmental perturbations can both cause de-canalization of the phenotype [56]. Yet, there is limited experimental evidence whether these two sources of variation also affect the same elements of developmental systems. When comparing the phenotypic effects of mutational vs. environmental perturbation, analyses are often restricted to a single or few environmental conditions using a single or few genetic variants. MA lines provide a more extensive and unbiased sampling of genotypic space. Yet, unlike mutation, environments cannot be systematically sampled. We therefore limit our comparison to six environments examined in an earlier study [34], showing that certain vulval variants are specifically generated in certain environments and genotypes. Several of these previously observed variant patterns were also frequently found after MA. Specifically, vulval centering shift variants on P7.p were never found in *C. elegans* N2 MA lines, but occurred often in MA lines derived from the other three ancestral genotypes. Similarly, N2 never generated P7.p centering shifts under starvation stress, while *C. briggsae* showed increased and increased frequency of this variant pattern. Mutational perturbations therefore may mirror environmental perturbations, so that both sources of variation reveal the genotype-dependence of developmental bias.

### Bias in developmental mutability and evolutionary trends

Examination of different *Caenorhabditis* MA lines allows us to detect axes of high mutational variability in the vulval developmental system. Whether or not such high mutational variance translates into actual evolution then depends on selection. Some of these phenotypic axes of least resistance upon mutation may correspond to traits under purifying selection. In this case, the available mutational variance does not result in phenotypic evolution. For other variant types, however, the high mutational variance may correspond to phenotypic evolution observed in the species or among closely related species. In the *Caenorhabditis* genus, intra- and interspecific variation in vulval patterning traits is limited to the frequency of P3.p adopting a 3° versus 4° fate, and to a lesser extent that of P4.p [37],[39],[57]. For these two vulval phenotypes we also found the greatest mutational variance. The mutational bias and the evolutionary trend in the vulva system thus mainly affect the same trait. At a larger evolutionary scale, a similar match between mutational pattern and evolution is found in the *Oscheius* genus, but for vulva variants that concern the second round of 3° cell divisions (variants #10–11). In this case, the mutational variance in the occurrence of the second round of 3° cell divisions appears high in *Oscheius tipulae* CEW1 (from EMS-induced mutant lines) [58] and the same trait varies greatly within the *Oscheius* genus [24],[37],[39]. By contrast, we found very little mutational variation in the occurrence of a second division round for the 3° cells (variants #10–11), and these traits are invariant within the *Caenorhabditis* genus, presumably because of developmental constraints. Such studies of relative trait mutability are thus crucial to understand variation in evolutionary trends between taxa and thereby bridge the gap between micro- and macro-evolutionary variation.

In conclusion, our results provide an empirical view on the developmental variation induced by spontaneous random mutation. In the case of the highly canalized vulval developmental system, this variation is generally very subtle and difficult to quantify. In addition, the induced phenotypic variation is very complex despite the seeming molecular and developmental simplicity of this process. Nonetheless, we could uncover a number of developmental and genetic biases in the introduction of phenotypic variation, supporting the notion that such asymmetries bias the range of phenotypes available for selection to act upon [11]–[15],[18],[19]. Many more studies characterizing biases in the production spontaneous phenotypic variation (and its correspondence to evolutionary variation of the studied phenotypes) are required to evaluate whether such asymmetries play important roles as direction-giving forces in the evolutionary process.

## Materials and Methods

### Mutation accumulation lines

The main set of mutation accumulation (MA) lines in this study is that of Baer et al. [25] (called CFB lines). The lines were originated from a single highly inbred individual from each of two isogenic wild isolates of *C. elegans* (N2 and PB306 isolates) and *C. briggsae* (HK104 and PB800 isolates). Criteria for choice of these isolates are given in [25]. The mutation accumulation experiments began with 100 replicate MA lines per isolate. Details of the mutation accumulation protocols are given in the original paper. Briefly, highly inbred stocks of each isolate were replicated 100 times and perpetuated by single-hermaphrodite transfer for 250 generations. This protocol results in a genetic effective population size of *N_e_*≈1 (the approximation is the result of occasionally having to use backup stocks of worms when the original worm did not survive), thereby minimizing the efficiency of natural selection and ensuring that all but the most deleterious mutations behave according to neutral dynamics. Worm stocks, including G_0_ ancestral controls and ultimate generation MA lines, were cryopreserved using standard methods [59].

### Wild isolates

Wild isolates of *C. elegans* (N = 25) and *C. briggsae* (N = 10) used in this study are listed in Table S6. Both species display a high selfing rate in natural populations [52],[60]. The (isogenic) wild isolates were originally established by selfing populations derived from a single individual isolated from the wild.

### Scoring of vulval cell fates

Worms were kept on Petri dishes (55 mm diameter) filled with NGM (Nematode Growth Medium) agar, seeded with approximately 200 µl bacterial suspension of the *E. coli* strain OP50. All experiments were carried out at 20°C. For each of three experimental blocks, a random set of MA lines and the four ancestral controls were thawed (for samples size, see below). To eliminate potential genetic variation in the stock culture, a single individual from each line was selected to initiate the experimental populations. After population expansion, 20–30 adult hermaphrodites per line were hypochlorite treated to clear individuals form potential microbial contaminations. (At this time, for each of the four ancestral controls, multiple replicates were established except for the first block). The resulting eggs were allowed to develop into adults at which stage 20 hermaphrodites (from the same NGM plate) were transferred to a new NGM plate. When the majority of the offspring had reached the L4 stage (after approximately 2–5 days depending on the line), 50 offspring/line were randomly selected to score their vulval phenotype. The vulval cell phenotype was determined during the early to mid L4 stage using Nomarski microscopy on individuals anaesthetized with sodium azide [59]. We counted induced cells and determined the fates of the cells P3.p to P8.p as described previously [44]. MA and control lines underwent approximately 4–6 generations on NGM plates (at low densities) between thawing and scoring.

We defined different types of vulval developmental variants (shown in Figure 2) by taking into account developmental features of the system. Note that due to replacement regulation between vulval precursor cells [31], the fate of each individual cell is not independent from that of the other cells. For example, when the anchor cell is positioned on P5.p, the entire pattern is displaced anteriorly and four Pn.p cell fates are affected simultaneously; if P5.p is missing, P4.p adopts a 2° fate; if the anchor cell is missing, the fates of P(5–7).p switch to a 3° fate, etc. Defining 14 distinct variant types allowed us to greatly lower the number of variant types compared to the combination of each fate for each cell (1°/2°/3°/4°/missing x 6 = 30 classes). Some of these variants correspond to changes due to independent developmental events as defined by mutational analysis [24],[53],[61]. For example, hypoinduction phenotypes through cell fate change from a vulval fate to a non-vulval fate (trait #2) likely occur through low activities of Ras or possibly Wnt pathways (Induction Vulvaless in [61]). In contrast, hypoinduction phenotypes arising by lack of Pn.p cells (trait #3) occur because of cell death or earlier switch in cell fate (Generation Vulvaless in [61]).

### Sample sizes

The following number of MA and control lines were analyzed for each isolate: HK104 (44 MA lines, 17 control lines), PB800 (53 MA lines, 17 control lines), PB306 (51 MA lines, 17 control lines) and N2 (52 MA lines, 17 control lines). For each MA and control line, 50 individuals were scored for their vulval phenotype.

### Data analysis: MA lines

There are two fundamental observable quantities of interest in a MA experiment—the change in the trait mean and the change in the variance. In this study, vulval character state is a binary random variable X with state 0 = wild-type and state 1 = non-canonical” (for traits 1–13). The data are binomially-distributed with parameter *p* = Pr(X = 1). Within a genotype/treatment group (“treatment” = MA or G_0_ ancestral control), each line provides a single independent estimate of *p*.

#### (i) Change in the mean (*R_m_*)

The per-generation change in the trait mean can be considered either on the raw scale (*R_m_*, the slope of the regression of the trait value against time, measured in generations of MA) or scaled as a fraction of the generation 0 mean (*ΔM = R_m_/M*_0_, where *M_0_* is the ancestral mean). *ΔM* is typically the more meaningful of the two because the average mutational effect is meaningful only relative to the starting phenotype, but the interpretation of *ΔM* breaks down when *M_0_* is close to zero. In the extreme case of a mutation that increases the frequency of a variant phenotype from 0 to 1/*n*, *ΔM* is infinite for all *n*. In this study we use *R_m_* as the measure of the change in the frequency of variant vulva phenotypes because of the very low frequency of variant phenotypes in the ancestral *C. elegans* controls.

We first tested for an effect of assay block using a general linear mixed model as implemented in SAS v. 9.2 PROC GLIMMIX, testing each isolate individually and employing a logit link function (http://support.sas.com/rnd/app/papers/glimmix.pdf). Block, treatment (MA vs. control) and their interaction are considered fixed effects; significance of approximate F-tests for fixed effects is determined by the residual pseudo-likelihood method [62]; error degrees of freedom are calculated by the Kenward-Rogers method. The model is *p_T_ = Block + Treatment + Block x Treatment + error*, where *p_T_* is the binomial parameter. In no case was there a significant main effect of or interaction with block (P>0.1 in all cases), so data were pooled over blocks for subsequent analyses.

To assess the statistical significance of differences between groups in *R_m_*, we used a bootstrap resampling protocol, as follows. A pseudo-dataset was constructed by resampling the data with replacement at the level of line, maintaining the same number of control and MA lines as in the original data set. The mean binomial parameter *p* was calculated for control and MA lines separately and *R_m_* estimated as *(p_MA_-p_0_)/t*, where *t* is the number of generations of MA. This procedure was repeated 1000 times; the upper and lower 2.5% of pseudo-estimates establish approximate 95% confidence limits on *R_m_* [63]. Differences between groups are considered significant if the 95% confidence intervals do not overlap.

To investigate the possibility that the variation among traits in *R_m_* may vary between species and/or isolates - that is, that there is a trait x taxon (here species or isolate) interaction in the variable *R_m_* - we employed a general linear mixed model as implemented in SAS. v. 9.2 PROC MIXED. We first calculated a line-specific value of *R_m_* for each trait *j* for each MA line *i* (*R_m,i_*_j_) by subtracting the control mean value of *p* from each line-specific value of *p*, i.e., *R_m,ji_* = *p_ ij_ −p̂_0,j_* (we omit the number of MA generations, *t*, from this calculation for convenience). We then analyzed the linear model *R_m_ = Species + Trait + Trait x Species + Isolate(Species) + Trait x Isolate(Species) + Error*. Six of the 3000 data points were identified as high outliers by visual inspection of a Q-Q plot and removed from the analysis. Residual (error) variance was estimated separately for each trait/species combination via the GROUP option in PROC MIXED; the model failed to converge when residuals were estimated for each trait/isolate combination.

The above analysis is potentially compromised in two ways. First, the analysis is strictly valid only when the data are normally distributed; the data in this case deviate substantially from normality and cannot be transformed to meet the assumption of normality. To assess the sensitivity of the analysis to violation of distributional assumptions we performed randomization tests using the aforementioned linear model, with data randomly permuted over traits within each isolate. If the test is robust, the frequency of a particular outcome in randomly permuted data should be approximately equivalent to its theoretical probability of occurrence given the assumptions (i.e., its P-value). In every case we examined, the distribution of P-values was almost identical to the theoretical expectation.

Second, the analysis treats the control mean for each trait/isolate combination as a parameter of the model rather than a random variable. Therefore, the P-values associated with the pseudo-F-tests [62] are inflated to some degree. We report “nominal” P-values, which are useful for comparison of the relative magnitudes of the effects within the model but cannot be taken at face value. However, estimates of control means are based on many more measurements (usually 17 times, i.e. 850 individuals) than estimates for any given MA line (50 individuals), so the sampling variance of the control mean should be much less than the within-line variance of any MA line.

#### (ii) Change in the among-line variance *(ΔV)*

The mutational variance, *V_M_*, is typically estimated from the per-generation change in the among-line component of variance [64]. However, it is not possible to estimate meaningful within- and among-line components of variance from these MA data because we have only a single independent estimate from each line and the within-line variance *[ = p(1-p)]* is a function of the mean (*p*). Instead, we consider the change in the variance in line means, i.e., the variance in the binomial parameter *p_j_*, where *p_j_* is the frequency of variant phenotypes in line *j*. The change in the variance is calculated as Δ*V = (V_MA_−V_0_)/t*, where V_MA_ is the variance in *p* among MA line means, *V_0_* is the variance in *p* among ancestral control line means, and *t* is the number of generations of MA. If the ancestral control is homozygous at all loci as assumed, *V_0_* provides an estimate of the within-line variance and *ΔV/*2 provides an estimate of the mutational variance. We use the term *ΔV* rather than *V_M_* to emphasize that the per-generation increase in variance is not calculated from variance components. Note that although the within-line variance is a defined function of the mean *[ = p(1-p)]*, the variance in line means is not. Differences among groups in *ΔV* were assessed using the same bootstrap protocol as described above for *R_m_*. For each pseudo-dataset we calculated the variance in *p* for control and MA lines and then calculated *ΔV*. Confidence intervals and significance criterion for *ΔV* were determined as for *R_m_*.

#### (iii) Mutational correlations

We estimated mutational correlations of vulval development (*p*) with two fitness-related traits that we previously assayed in these MA lines [28],[45]. The first trait is lifetime reproductive output (called “Total Fitness”, *W*, in [28]), which is closely correlated with demographic fitness (Pearson's *r*≈0.9; Baer, unpublished data). The second trait is the environmental (here meant as within-line) coefficient of variation in *W* (*CV_E,W_*), which provides an estimate of environmental canalization of *W* [45]. Because we cannot estimate (co)variance components for vulval development, we report correlations calculated from (co)variances of line means, which will generally underestimate the absolute value of the among-line correlation [29]. *W* and *CV_E,W_* are not independent so we do not report the correlation between those variables.

To accommodate among-block variation in *W* and *CV_E,W_*, we first defined a new variable *w_ijk_* as the proportional deviation of an individual worm's *W* from the block mean *W* of the ancestral control, i.e., 
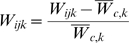
, where *W_ijk_* is the value of individual *i* in MA line *j* in experimental block *k* and 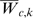 is the mean of the ancestral controls in block *k*. We next calculated line means 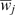 and within-line CVs, *CV_E,j_*. We then estimated the (co)variances of *p_j_* and *w_j_* and *CV_E, j_*. Finally, we calculated a corrected correlation 
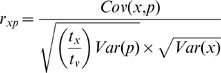
, where *x* represents the relevant variable (*w_j_* or *CV_E,j_*), *t_x_* is the number of MA generations at the time *x* was measured and *t_v_* is the number of MA generations at the time vulval development was measured. The term 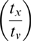 enters the denominator because, if two traits are measured at different generations, mutations that occur after the first trait was measured cannot contribute to the correlation between the two traits; Var(*p*) is multiplied by this fraction - rather than Var(*w*) - because vulval development was measured at a later generation than was fitness. Fitness variables were measured at 200 and 220 generations; we used the average value of 210 generations for *t_x_*. (Co)variances were estimated by REML using SAS v. 9.2 PROC MIXED with unstructured covariance (TYPE = UN option).

### Data analysis: wild isolates

If wild isolates are homozygous at all loci (a plausible approximation for a highly-selfing species; see above), the standing genetic variance (*V_G_*) can be estimated from the among-line component of variance [65]. However, for 22/25 wild isolates of *C. elegans*, we only have a single estimate of the binomial parameter *p* and therefore cannot meaningfully partition the variance in *p* into within and among-isolate components. Instead, we use the variance in isolate means *V_ L̂_* as an upper bound on *V_G_*. Using Δ*V* and *V_ L̂_* to approximate the mutational variance *V_M_* and *V_G_*, respectively, the relationship *V_G_*≈*V_M_*/*S* provides an estimate of the strength of selection against new mutations (*S*), provided the system is at mutation-(purifying) selection balance (MSB) [47]. For the isolates for which we have multiple independent estimates of *p*, we partitioned the variance into within- and among-isolate components using REML as implemented in the MIXED procedure of SAS v. 9.2. We can then compare the variance components of these isolates to *V_ L̂_* to gain a rough idea of the relative fraction of the variance that is among isolates.

To establish confidence intervals on Δ*V* and *V_ L̂_* we used a delete-one jackknife method [66] to estimate the standard error of the statistic, which was then used in the standard Student's-t calculation of the 95% confidence limits [67],

### Ras pathway activity measurements using transcriptional reporter *egl-17::cfp*

To estimate Ras pathway activity level in the *C. elegans* N2 and PB306 isolates, we used a previously generated transgenic strain containing an integrated transcriptional reporter construct for the LET-60/Ras pathway, *egl-17::cfp-lacZ* (strain GS3582) [41]. This construct contains a nuclear localization sequences upstream of the CFP coding sequence and was generated using the isolate N2 [41]. We then generated the *egl-17::cfp-lacZ* strain JU480 from the strain GS3582 by genetically removing the transformation marker *unc-4(e120)*. Each integrated transgenic array generated in the N2 background was outcrossed ten times to PB306, by crossing at each generation the male progeny to wild hermaphrodites. After ten backcrosses, the introgressed line was made isogenic by selfing for several generations, yielding strain JU488.

The CFP fluorescence quantification experiment was performed as described in [34] in standard conditions at 20°C, for JU480 and JU488 in parallel. For each individual/image, we quantified signal (pixel) intensity of P5.p, P6.p and P7.p. For each examined developmental stage, we carried out an ANOVA (JMP 7.0 for Mac) testing for the fixed effects of isolate, individual (nested in isolate), cell, and the interaction between isolate and cell type using mean signal intensity as a response variable. The inclusion of the effect individual(isolate) allowed us to control for the non-independence between measures of P5.p, P6.p, and P7.p taken from a single individual. Post-hoc tests (Tukey's HSD) were then performed to determine differences in signal expression between isolates and cells (P5.p. P6.p, P7.p).

## Supporting Information

Table S1 Data table. Raw data set for CFB lines, with each row corresponding to an individual worm (worksheet “data)”; for data coding see worksheet “abbreviations”.
(2.96 MB XLS)

Table S2 Per-generation change in the frequency of variant phenotypes, *R_m_*. Classes and traits are defined in the text. Tabled values are the actual value multiplied by 10^5^ in (A,B), by 10^3^ in (C), and 10^2^ in (D); standard errors of the mean are in parentheses except for “Total proportion” in which the 95% confidence intervals are presented. The same analysis is presented graphically in Figure 3 for the 14 traits. Sample Sizes: HK104 (44 MA lines, 17 control lines), PB800 (53 MA lines, 17 control lines), PB306 (51 MA lines, 17 control lines) and N2 (52 MA lines, 17 control lines). For each MA and control line, 50 individuals were scored for their vulval phenotype.
(0.05 MB DOC)

Table S3 Per-generation change in the variance among line means, ΔV. SEM are in parentheses. Categories are defined in the text. “E-n” represents 10^-nth^ power. For sample sizes, see legend.
(0.05 MB DOC)

Table S4 Mutational Correlations. Cell entries are the correlation of MA line means between variables in the row/column. Abbreviations are: Class A variants (#1–4); Class B+C variants (#5–13); *CV_E,W_*, within-line coefficient of variation in lifetime fecundity [Baer CF (2008) Am Nat 172: 272–281]; *W*, lifetime fecundity (including 0s) [Baer CF et al. (2005) Proc Natl Acad Sci USA 102: 5785–5790]. * p<0.05, ** p<0.01, *** p<0.001. For sample sizes, see legend Table S2.
(0.04 MB DOC)

Table S5 Mixed model interaction results. This analysis omits P3.p 3 and includes all data (no outliers removed). Error variance was estimated separately for each trait/species combination. Num: Numerator. Den: Denominator. For sample sizes, see legend Table S2.
(0.03 MB DOC)

Table S6 Observations of vulval developmental variants in wild isolates. N: number of animals (total and each class of variant). See Main Table 1 for explanation of variant categories (A: Variants with disrupted 2°−1°−2° pattern), (B: Variants with complete 2°−1°−2° pattern) (C: Adoption of 4° fate by P4.p and P8.p). Only one wild isolate per sampling location is reported here.
(0.10 MB DOC)

Table S7 Results of statistical tests for comparison of Ras pathway activity in ancestral isolates of *C. elegans* (N2 versus PB306) using the *egl-17::cfp-lacZ* reporter. For each developmental stage, we carried out an ANOVA (JMP 7.0) testing for the fixed effects of *environment*, *individual(environment)*, *cell*, and the interaction between *environment* and *cell* using mean signal intensity as a response variable. The inclusion of the effect *individual(environment)* allowed us to control for the non-independence between measures of P5.p, P6.p, and P7.p taken from a single individual.
(0.05 MB DOC)
