# Supplementary material for: Bias and Evolution of the Mutationally Accessible Phenotypic Space in a Developmental System
Source: PLoS Genet. 2010 Mar 12;6(3):e1000877. doi: 10.1371/journal.pgen.1000877 (PMC2837400; doi:10.1371/journal.pgen.1000877)
Supplement: Table S2 — Per-generation change in the frequency of variant phenotypes, Rm. Classes and traits are defined in the text. Tabled values are the actual value multiplied by 105 in (A,B), by 103 in (C), and 102 in (D); standard errors of the mean are in parentheses except for “Total proportion” in which the 95% confidence intervals are presented. The same analysis is presented graphically in Figure 3 for the 14 traits. Sample Sizes: HK104 (44 MA lines, 17 control lines), PB800 (53 MA lines, 17 control lines), PB306 (51 MA lines, 17 control lines) and N2 (52 MA lines, 17 control lines). For each MA and control line, 50 individuals were scored for their vulval phenotype. (0.05 MB DOC) [file pgen.1000877.s002.doc]

**Table S2**

Class A: Variants with disrupted 2°-1°-2° pattern (defects)

| Species | ***C. briggsae*** | | ***C. elegans*** | |
| --- | --- | --- | --- | --- |
| Isolate | **HK104** | **PB800** | **N2** | **PB306** |
| 1. Hyperinduction | 2.41 (1.53) | 1.67 (0.75) | 0.46 (0.26) | -0.35 (0.81) |
| 2. Hypoinduction  (3° or 4° cell fate) | 0.98 (0.80) | 3.37 (1.65) | 0.62 (0.30) | 0.16 (0.15) |
| 3. Hypoinduction (missing cells) | 0.77 (0.45) | 0.31 (0.22) | 0 | 0.15 (0.15) |
| 4. Other fate misspecification | 2.52 (2.34) | 0.31 (0.31) | 1.10 (0.36) | 0.14 (0.61) |
| Total (A) | 4.27 (0.51, 10.44) | 4.76 (1.06, 9.81) | 2.02 (1.08, 3.08) | 0.10 (-2.51, 2.20) |

Class B: Variants with complete 2°-1°-2° pattern

| 5. Hyperinduction | 0 | 0.76 (0.43) | 0.15 (0.15) | 2.83 (1.51) |
| --- | --- | --- | --- | --- |
| **Vulval centering shifts:** | 4.69 (2.57) | 3.04 (1.32) | 2.81 (1.52) | 1.77 (1.04) |
| 6. Centering on P5.p | 3.66 (2.16) | 1.22 (0.60) | 2.81 (1.52) | 1.61 (1.01) |
| 7. Centering on P7.p | 1.04 (1.43) | 1.83 (1.18) | 0 | 0.16 (0.15) |
| **Missing Pn.p cells:** | 2.49 (1.07) | 3.14 (1.43) | 1.19 (0.94) | 1.75 (0.94) |
| 8. Anterior cell missing | 0.58 (0.40) | 0.74 (0.70) | -1.18 (0.57) | 0.31 (0.61) |
| 9. Posterior cell missing | 1.91 (0.78) | 2.41 (1.09) | 1.37 (0.57) | 1.45 (0.61) |
| **Extra cell divisions:** | 0.48 (1.67) | 0.78 (0.39) | 0.76 (0.34) | 1.44 (0.79) |
| 10. Anterior 3°cell | 0.44 (1.19) | 0.31 (0.21) | 0.31 (0.21) | 0.66 (0.43) |
| 1I. Posterior 3° cell | 0.04 (0.55) | 0.47 (0.26) | 0.46 (0.26) | 0.78 (0.48) |
| Total (B) | 10.07 (3.85, 16.84) | 7.73 (3.30, 13.13) | 4.91 (1.22, 9.50) | 7.79 (3.14, 13.33) |

**Class C: Adoption of 4° fate by P4.p and P8.p**

| 12. P4.p: 4° fate | 3.99 (2.01) | 8.67 (2.97) | 2.08 (1.15) | 0.25 (0.88) |
| --- | --- | --- | --- | --- |
| 13. P8.p: 4° fate | 3.74 (2.56) | -3.09 (3.78) | 0 | 0.15 (0.61) |

Class D: Adoption of 4° fate by P3.p

| 14. P3.p: 4° fate | 20.87 (7.36) | 12.67 (4.80) | - 2.62 (11.23) | 34.91 (10.22) |
| --- | --- | --- | --- | --- |

.
