# Supplementary material for: Bias and Evolution of the Mutationally Accessible Phenotypic Space in a Developmental System
Source: PLoS Genet. 2010 Mar 12;6(3):e1000877. doi: 10.1371/journal.pgen.1000877 (PMC2837400; doi:10.1371/journal.pgen.1000877)
Supplement: Table S3 — Per-generation change in the variance among line means, ΔV. SEM are in parentheses. Categories are defined in the text. “E-n” represents 10-nth power. For sample sizes, see legend. (0.05 MB DOC) [file pgen.1000877.s003.doc]

**Table S3**

**Class A: Variants with disrupted 2°-1°-2° pattern (defects)**

| Species | ***C. briggsae*** | | ***C. elegans*** | |
| --- | --- | --- | --- | --- |
| Isolate | **HK104** | **PB800** | **N2** | **PB306** |
| 1. Hyperinduction | 0 | 1.18 E-7 (8.69 E-8) | 1.87 E-8 (1.61 E-8) | 1.20 E-8 (6.83 E-8) |
| 2. Hypoinduction  (3° or 4° cell fate) | 1.73 E-7 (1.32 E-7) | 2.04 E-6 (1.11 E-6) | 6.72 E-8 (2.71 E-8) | 8.99 E-9 (1.19E-8) |
| 3. Hypoinduction (missing cells) | 1.07 E-7 (6.60 E-8) | 3.51 E-8 (2.00 E-8) | 0 | 1.76 E-8 (1.58 E-8) |
| 4. Other fate misspecification | 2.94 E-6 (2.62 E-6) | 7.14 E-8 (6.47 E-8) | 8.26 E-8 (2.83 E-8) | -3.54 E-8 (4.64 E-8) |
| Total (A) | 3.55 E-6 (2.93 E-6) | 4.27 E-6 (2.61 E-6) | 2.19 E-7 (7.86 E-8) | -9.08 E-8 (1.86 E-7) |

**Class B: Variants with complete 2°-1°-2° pattern**

| 5. Hyperinduction | 1.23 E-6 (1.01 E-6) | 1.18 E-7 (6.30 E-8) | 1.87 E-8 (1.61 E-8) | 1.54 E-6 (1.28 E-6) |
| --- | --- | --- | --- | --- |
| **Centering shifts:** | 3.06 E-6 (1.78 E-6) | 4.56 E-7 (2.38 E-7) | 6.15 E-7 (3.19 E-7) | 7.17 E-7 (5.68 E-7) |
| 6. Centering on P5.p | 2.28 E-6 (1.79 E-6) | 2.70 E-7 (1.39 E-7) | 6.15 E-7 (3.19 E-7) | 6.94 E-7 (5.69 E-7) |
| 7. Centering on P7.p | 9.02 E-7 (8.05 E-7) | 6.69 E-8 (2.84 E-8) | 0 | 8.99 E-9 (1.19 E-8) |
| **Missing Pn.p cells:** | 6.25 E-7 (3.94 E-7) | 1.26 E-6 (9.35 E-7) | 3.94 E-7 (2.64 E-7) | 3.93 E-7 (2.27 E-7) |
| 8. Anterior cell | 9.12 E-8 (6.73 E-8) | 1.38 E-7 (8.37 E-7) | -1.14 E-8 (7.04 E-8) | 3.66 E-8 (4.91 E-8) |
| 9. Posterior cell | 3.30 E-7 (1.46 E-7) | 7.49 E-7 (5.46 E-7) | 3.75 E-7 (2.52 E-7) | 2.79 E-7 (1.43 E-7) |
| **Extra cell divisions:** | -3.45 E-8 (4.08 E-7) | 1.05 E-7 (6.55 E-8) | 5.23 E-8 (2.73 E-8) | 2.89 E-7 (1.91 E-7) |
| 10. Anterior 3°cell | 5.03 E-8 (1.05 E-7) | 3.56 E-8 (2.11 E-8) | 3.48 E-8 (2.27 E-8) | 1.05 E-7 (7.47 E-8) |
| 1I. Posterior 3° cell | 5.26 E-9 (4.53 E-8) | 3.53 E-8 (2.23 E-8) | 1.83 E-8 (1.63 E-8) | 1.22 E-7 (7.46 E-8) |
| Total (B) | 4.31 E-6 (1.71 E-6) | 2.87 E-6 (1.32 E-6) | 1.61 E-6 (5.97 E-6) | 4.06 E-6 (1.89 E-6) |

**Class C: Adoption of 4° fate by P4.p and P8.p**

| 12. P4.p: 4° fate | 1.37 E-6 (6.06 E-7) | 3.29 E-6 (1.60 E-6) | 5.83 E-7 (3.75 E-7) | 6.13 E-8 (6.92 E-8) |
| --- | --- | --- | --- | --- |
| 13. P8.p: 4° fate | 1.17 E-6 (3.73 E-7) | 3.95 E-6 (1.72 E-6) | 0 | 2.32 E-8 (6.46 E-8) |

**Class D:** Adoption of 4° fate by P3.p

| 14. P3.p: 4° fate | 2.34 E-5 (1.57 E-5) | 7.49 E-6 (3.50 E-6) | 1.35 E-5 (1.05 E-5) | 3.91 E-5 (1.13 E-5) |
| --- | --- | --- | --- | --- |
