# Supplementary material for: Bias and Evolution of the Mutationally Accessible Phenotypic Space in a Developmental System
Source: PLoS Genet. 2010 Mar 12;6(3):e1000877. doi: 10.1371/journal.pgen.1000877 (PMC2837400; doi:10.1371/journal.pgen.1000877)
Supplement: Table S4 — Mutational Correlations. Cell entries are the correlation of MA line means between variables in the row/column. Abbreviations are: Class A variants (#1–4); Class B+C variants (#5–13); CVE,W, within-line coefficient of variation in lifetime fecundity [Baer CF (2008) Am Nat 172: 272–281]; W, lifetime fecundity (including 0s) [Baer CF et al. (2005) Proc Natl Acad Sci USA 102: 5785–5790]. * p<0.05, ** p<0.01, *** p<0.001. For sample sizes, see legend Table S2. (0.04 MB DOC) [file pgen.1000877.s004.doc]

**Table S4**

| Isolate/Species |  | Variant | *CV_E,W_* | *W* |
| --- | --- | --- | --- | --- |
|  |  |  |  |  |
| HK104/*C.br*. | Class A | 0.26 | -0.09 | -0.10 |
|  | Class B+C | - | 0.09 | -0.24 |
|  |  |  |  |  |
| PB800/*C.br*. | Class A | 0.74** | -0.10 | 0.14 |
|  | Class B+C | - | 0.14 | -0.09 |
|  |  |  |  |  |
| N2/*C.el*. | Class A | 0.22 | -0.11 | 0.11 |
|  | Class B+C | - | 0.48** | -0.61*** |
|  |  |  |  |  |
| PB306/*C.el*. | Class A | 0.51*** | 0.49** | -0.35 |
|  | Class B+C | - | 0.48*** | -0.36* |
