# Supplementary material for: Bias and Evolution of the Mutationally Accessible Phenotypic Space in a Developmental System
Source: PLoS Genet. 2010 Mar 12;6(3):e1000877. doi: 10.1371/journal.pgen.1000877 (PMC2837400; doi:10.1371/journal.pgen.1000877)
Supplement: Table S5 — Mixed model interaction results. This analysis omits P3.p 3 and includes all data (no outliers removed). Error variance was estimated separately for each trait/species combination. Num: Numerator. Den: Denominator. For sample sizes, see legend Table S2. (0.03 MB DOC) [file pgen.1000877.s005.doc]

**Table S5**

| **Effect** | **Num DF** | **Den DF** | **F Value** | **Pr > F** |
| --- | --- | --- | --- | --- |
| Trait | 12 | 664 | 3.63 | <.0001 |
| Species | 1 | 823 | 8.98 | 0.0028 |
| Species*Trait | 12 | 664 | 1.39 | 0.1655 |
| Isolate(Species) | 2 | 702 | 0.14 | 0.8687 |
| Isolate*Trait(Species) | 24 | 887 | 1.32 | 0.1414 |
