# Supplementary material for: Bias and Evolution of the Mutationally Accessible Phenotypic Space in a Developmental System
Source: PLoS Genet. 2010 Mar 12;6(3):e1000877. doi: 10.1371/journal.pgen.1000877 (PMC2837400; doi:10.1371/journal.pgen.1000877)
Supplement: Table S6 — Observations of vulval developmental variants in wild isolates. N: number of animals (total and each class of variant). See Main Table 1 for explanation of variant categories (A: Variants with disrupted 2°−1°−2° pattern), (B: Variants with complete 2°−1°−2° pattern) (C: Adoption of 4° fate by P4.p and P8.p). Only one wild isolate per sampling location is reported here. (0.10 MB DOC) [file pgen.1000877.s006.doc]

**Table S6**

| **Species** | **Isolate** | **Experiment** | **N** | **N (A)** | **N (B)** | **N (C)** | **Reference** |
| --- | --- | --- | --- | --- | --- | --- | --- |
| *C. briggsae* | AF16 | 24 | 100 | 0 | 1 | 12 | Braendle & Félix 2008 |
| *C. briggsae* | AF16 | 25 | 100 | 0 | 0 | 8 | Braendle & Félix 2008 |
| *C. briggsae* | AF16 | 26 | 100 | 0 | 0 | 6 | Braendle & Félix 2008 |
| *C. briggsae* | ED3082 | 44 | 100 | 0 | 0 | 23 | Félix, unpublished |
| *C. briggsae* | ED3092 | 44 | 100 | 1 | 0 | 3 | Félix, unpublished |
| *C. briggsae* | HK104 | 24 | 100 | 0 | 0 | 6 | Braendle & Félix 2008 |
| *C. briggsae* | HK104 | 25 | 100 | 0 | 1 | 3 | Braendle & Félix 2008 |
| *C. briggsae* | HK104 | 26 | 100 | 0 | 0 | 3 | Braendle & Félix 2008 |
| *C. briggsae* | JU439 | 24 | 100 | 0 | 0 | 3 | Braendle & Félix 2008 |
| *C. briggsae* | JU439 | 25 | 100 | 1 | 0 | 1 | Braendle & Félix 2008 |
| *C. briggsae* | JU439 | 26 | 100 | 0 | 1 | 1 | Braendle & Félix 2008 |
| *C. briggsae* | JU441 | 42 | 115 | 0 | 1 | 6 | Félix, unpublished |
| *C. briggsae* | JU516 | 42 | 105 | 0 | 0 | 0 | Félix, unpublished |
| *C. briggsae* | JU725 | 43 | 111 | 1 | 0 | 3 | Dolgin et al. 2008; Félix, unpublished |
| *C. briggsae* | JU726 | 43 | 102 | 0 | 0 | 7 | Dolgin et al. 2008; Félix, unpublished |
| *C. briggsae* | PB826 | 36 | 100 | 0 | 0 | 5 | Delattre & Félix 2001 |
| *C. elegans* | AB1 | 29 | 100 | 0 | 0 | 0 | Braendle, unpublished |
| *C. elegans* | AB4 | 40 | 143 | 0 | 1 | 0 | Félix, unpublished |
| *C. elegans* | CB3191 | 40 | 142 | 0 | 0 | 0 | Félix, unpublished |
| *C. elegans* | CB4852 | 40 | 145 | 0 | 0 | 0 | Félix, unpublished |
| *C. elegans* | CB4853 | 40 | 180 | 1 | 2 | 1 | Félix, unpublished |
| *C. elegans* | CB4854 | 39 | 147 | 1 | 0 | 1 | Félix, unpublished |
| *C. elegans* | CB4855 | 31 | 238 | 0 | 0 | 0 | Delattre & Félix 2001 |
| *C. elegans* | CB4856 | 15 | 100 | 0 | 0 | 0 | Braendle & Félix 2008 |
| *C. elegans* | CB4856 | 16 | 100 | 0 | 0 | 0 | Braendle & Félix 2008 |
| *C. elegans* | CB4856 | 17 | 100 | 0 | 0 | 0 | Braendle & Félix 2008 |
| *C. elegans* | CB4856 | 28 | 97 | 0 | 0 | 0 | Braendle, unpublished |
| *C. elegans* | CB4857 | 32 | 204 | 0 | 0 | 3 | Delattre & Félix 2001 |
| *C. elegans* | CB4932 | 33 | 201 | 0 | 1 | 0 | Delattre & Félix 2001 |
| *C. elegans* | DH424 | 35 | 103 | 0 | 1 | 1 | Delattre & Félix 2001 |
| *C. elegans* | ED3040 | 44 | 100 | 0 | 0 | 4 | Dolgin et al. 2008; Félix, unpublished |
| *C. elegans* | ED3046 | 44 | 100 | 0 | 0 | 0 | Dolgin et al. 2008; Félix, unpublished |
| *C. elegans* | ED3054 | 44 | 100 | 0 | 0 | 1 | Dolgin et al. 2008; Félix, unpublished |
| *C. elegans* | ED3077 | 44 | 100 | 0 | 0 | 2 | Dolgin et al. 2008; Félix, unpublished |
| *C. elegans* | JU258 | 10 | 112 | 1 | 0 | 0 | Braendle & Félix 2008 |
| *C. elegans* | JU258 | 11 | 81 | 0 | 0 | 0 | Braendle & Félix 2008 |
| *C. elegans* | JU258 | 12 | 157 | 0 | 2 | 0 | Braendle & Félix 2008 |
| *C. elegans* | JU258 | 13 | 53 | 0 | 0 | 0 | Braendle & Félix 2008 |
| *C. elegans* | JU258 | 14 | 97 | 0 | 0 | 0 | Braendle & Félix 2008 |
| *C. elegans* | JU258 | 18 | 100 | 0 | 0 | 1 | Braendle & Félix 2008 |
| *C. elegans* | JU258 | 19 | 100 | 0 | 3 | 0 | Braendle & Félix 2008 |
| *C. elegans* | JU258 | 20 | 100 | 1 | 0 | 0 | Braendle & Félix 2008 |
| *C. elegans* | JU262 | 37 | 182 | 0 | 0 | 6 | Félix, unpublished |
| *C. elegans* | JU319 | 41 | 119 | 0 | 0 | 0 | Félix, unpublished |
| *C. elegans* | KR314 | 34 | 199 | 0 | 0 | 1 | Delattre & Félix 2001 |
| *C. elegans* | LKC34 | 44 | 100 | 0 | 0 | 0 | Félix, unpublished |
| *C. elegans* | N2 | 1 | 112 | 0 | 1 | 0 | Braendle & Félix 2008 |
| *C. elegans* | N2 | 2 | 73 | 0 | 0 | 0 | Braendle & Félix 2008 |
| *C. elegans* | N2 | 3 | 116 | 0 | 0 | 0 | Braendle & Félix 2008 |
| *C. elegans* | N2 | 4 | 110 | 1 | 0 | 0 | Braendle & Félix 2008 |
| *C. elegans* | N2 | 5 | 97 | 0 | 1 | 0 | Braendle & Félix 2008 |
| *C. elegans* | N2 | 6 | 60 | 0 | 0 | 0 | Braendle & Félix 2008 |
| *C. elegans* | N2 | 7 | 242 | 2 | 0 | 1 | Braendle & Félix 2008 |
| *C. elegans* | N2 | 8 | 116 | 0 | 0 | 0 | Braendle & Félix 2008 |
| *C. elegans* | N2 | 9 | 74 | 0 | 0 | 0 | Braendle & Félix 2008 |
| *C. elegans* | N2 | 21 | 100 | 0 | 0 | 0 | Braendle & Félix 2008 |
| *C. elegans* | N2 | 22 | 100 | 0 | 0 | 0 | Braendle & Félix 2008 |
| *C. elegans* | N2 | 23 | 100 | 1 | 0 | 0 | Braendle & Félix 2008 |
| *C. elegans* | N2 | 30 | 1000 | 1 | 1 | 3 | Braendle & Félix 2008 |
| *C. elegans* | PB303 | 38 | 180 | 0 | 0 | 1 | Félix, unpublished |
| *C. elegans* | PB306 | 27 | 103 | 1 | 2 | 0 | Braendle, unpublished |
| *C. elegans* | PS2025 | 38 | 218 | 1 | 0 | 1 | Félix, unpublished |
| *C. elegans* | TR388 | 40 | 226 | 0 | 0 | 2 | Félix, unpublished |
