# Supplementary material for: Bias and Evolution of the Mutationally Accessible Phenotypic Space in a Developmental System
Source: PLoS Genet. 2010 Mar 12;6(3):e1000877. doi: 10.1371/journal.pgen.1000877 (PMC2837400; doi:10.1371/journal.pgen.1000877)
Supplement: Table S7 — Results of statistical tests for comparison of Ras pathway activity in ancestral isolates of C. elegans (N2 versus PB306) using the egl-17::cfp-lacZ reporter. For each developmental stage, we carried out an ANOVA (JMP 7.0) testing for the fixed effects of environment, individual(environment), cell, and the interaction between environment and cell using mean signal intensity as a response variable. The inclusion of the effect individual(environment) allowed us to control for the non-independence between measures of P5.p, P6.p, and P7.p taken from a single individual. (0.05 MB DOC) [file pgen.1000877.s007.doc]

**Table S7**

| **Mid L2** |  |  |  |  |
| --- | --- | --- | --- | --- |
|  |  |  |  |  |
| **Source** | **DF** | **SS** | **F** | **P** |
| Genotype | 1 | 70170.38 | 14.2289 | 0.0003 |
| Individual(Genotype) | 2 | 2070.73 | 0.2099 | 0.8109 |
| Cell | 2 | 659440.14 | 66.8594 | <.0001 |
| Cell x Genotype | 2 | 162511.41 | 16.4767 | <.0001 |
| Error | 118 | 581922.2 |  |  |
|  |  |  |  |  |
| **Lethargus L2/L3** |  |  |  |  |
|  |  |  |  |  |
| **Source** | **DF** | **SS** | **F** | **P** |
| Genotype | 1 | 2114.3 | 0.2655 | 0.6075 |
| Individual(Genotype) | 2 | 6734.5 | 0.4229 | 0.6563 |
| Cell | 2 | 2810754.1 | 176.4971 | <.0001 |
| Cell x Genotype | 2 | 19155.9 | 1.2029 | 0.3046 |
| Error | 100 | 796260.9 |  |  |
|  |  |  |  |  |
| **Early L3** |  |  |  |  |
|  |  |  |  |  |
| **Source** | **DF** | **SS** | **F** | **P** |
| Genotype | 1 | 47482.9 | 11.9854 | 0.0007 |
| Individual(Genotype) | 2 | 8944 | 1.1288 | 0.3261 |
| Cell | 2 | 7440453.8 | 939.0384 | <.0001 |
| Cell x Genotype | 2 | 19430.1 | 2.4522 | 0.0895 |
| Error | 154 | 610108.1 |  |  |
